# Levofloxacin-loaded surfactant nanocarriers: a computational study

**DOI:** 10.1039/d5na00884k

**Published:** 2026-01-24

**Authors:** Arin Sharoyan, Vahram Hakobyan, Hayk Melkonyan, Hrachya Ishkhanyan, Armen H. Poghosyan, Marine A. Parsadanyan, Ara P. Antonyan, Poghos O. Vardevanyan

**Affiliations:** a Department of Medical Laboratory, Division of Natural Sciences, Haigazian University Mexique, Kantari Street, 11-1748, Riad El Solh 11072090 Beirut Lebanon; b Central Clinical Military Hospital of the Ministry of Defense of RA 115 Muratsan 0008 Yerevan Armenia; c Computational Molecular Engineering Lab, Institute for Informatics and Automation Problems of NAS RA P. Sevak 1 0014 Yerevan Armenia; d Bioinformatics Laboratory, Institute for Informatics and Automation Problems of NAS RA P. Sevak 1 0014 Yerevan Armenia; e Laboratory of Biophysics of Sub-Cellular Structures, Research Institute of Biology, Yerevan State University A. Manoogian 1 0025 Yerevan Armenia poghosyan@gmail.com

## Abstract

We performed extensive all-atom molecular dynamics simulations to investigate the interaction dynamics and orientation of levofloxacin, a newer fluoroquinolone antibiotic, with anionic (SDS) and cationic (CTAB) micelles. The maximum drug/micelle ratio (loading capacity and entrapment efficiency) was estimated for anionic and cationic micelles. High encapsulation efficiencies were observed for SDS (average: ∼80%). In contrast, for CTAB micelles, the efficiency was ∼8%, indicating that the binding of levofloxacin molecules to SDS micelles is significantly higher than that to CTAB micelles. Tilted orientations were observed for levofloxacin in SDS micelles (∼48–51°) and in CTAB micelles (∼40–42°), where the positively charged piperazine group is anchored to anionic headgroups. In contrast, the negatively charged carboxylic group is close to cationic headgroups. Calculating the relative binding energies, we found that levofloxacin binds more strongly to SDS than CTAB. Due to π–π interactions and hydrogen bonding, the formation of concerted columnar stacks of levofloxacin was also recorded for both anionic and cationic micelles.

## Abstract 2

Molecular dynamics simulations reveal distinct binding modes of levofloxacin in anionic and cationic micelles, highlighting the impact of surfactant type on drug encapsulation and release dynamics.

## Graphical abstract

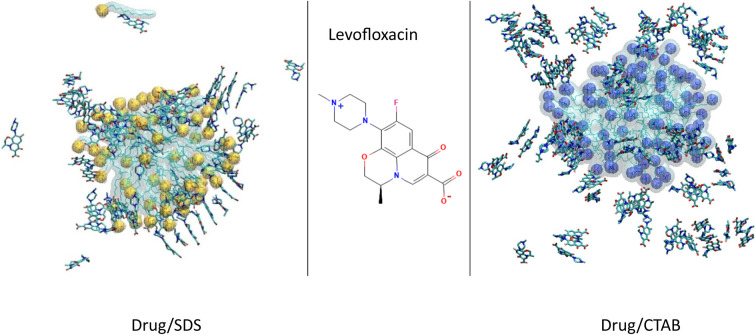

## Introduction

Nowadays, surfactants are widely used in drug delivery, as surfactant complexes (*e.g.*, micelles) can serve as delivery agents to mitigate drug side effects.^1–3^ Surfactants can encapsulate both hydrophobic and hydrophilic drugs, thereby protecting them from degradation. Different types of surfactant micelles (anionic, cationic, and neutral) can act as delivery nanocarriers for non-covalently bound drugs.^4^ Specifically, sodium dodecyl sulfate and trimethylammonium bromide micelles have been intensively used for this purpose.^5–11^

Levofloxacin is a third-generation fluoroquinolone antibiotic commonly used to treat infections of the respiratory tract, skin, urinary tract, and prostate.^12–14^ The interaction of fluoroquinolones with surfactants and the study of drug association with their micelles are crucial as a template for drug delivery systems. Therefore, the study of various fluoroquinolones (norfloxacin, ciprofloxacin, levofloxacin, *etc*.)/surfactant nanocarrier systems has recently attracted the interest of researchers, and various experimental^4,7,15–22^ and computational studies^22–24^ have been carried out. The fluoroquinolone drugs alter the surfactant critical micelle concentration (CMC), an important property from a pharmaceutical perspective, due to their ability to form micelle–drug delivery nanocarriers. Levofloxacin causes a decrease in the CMC of both anionic – sodium dodecyl sulfate (SDS)^7^ – and cationic – cetyltrimethylammonium bromide (CTAB)^23,25^ – micelles. In many studies mentioned above, attempts to enhance the fluoroquinolone solubility were made by leading to the accumulation of drug molecules, and among various types of surfactants, anionic SDS and cationic CTAB have been widely tested, as levofloxacin and its analogs with their zwitterionic form exhibit an affinity toward both cationic and anionic surfactants.^18,26,27^

However, besides the experimental data reported above, no direct computational study of the mentioned drugs with CTAB or SDS was found in the literature to reveal the accumulation properties and loading capacities of these systems. On the other hand, the orientational properties and binding affinity are also essential to figure out. From a methodical point of view, in line with real experiments, classic molecular dynamics (MD) studies are able to provide additional information and gain insight into the behavior of such a complex system.

In the current work, we aimed to study the effect of concentration on the solubilization of levofloxacin within anionic SDS and cationic CTAB micelles. We also focused on the loading capacity, entrapment efficiencies, binding affinity, and orientation of encapsulated levofloxacin drugs within the mentioned micelles.

## Construction and simulation details

**Fig. 1:**
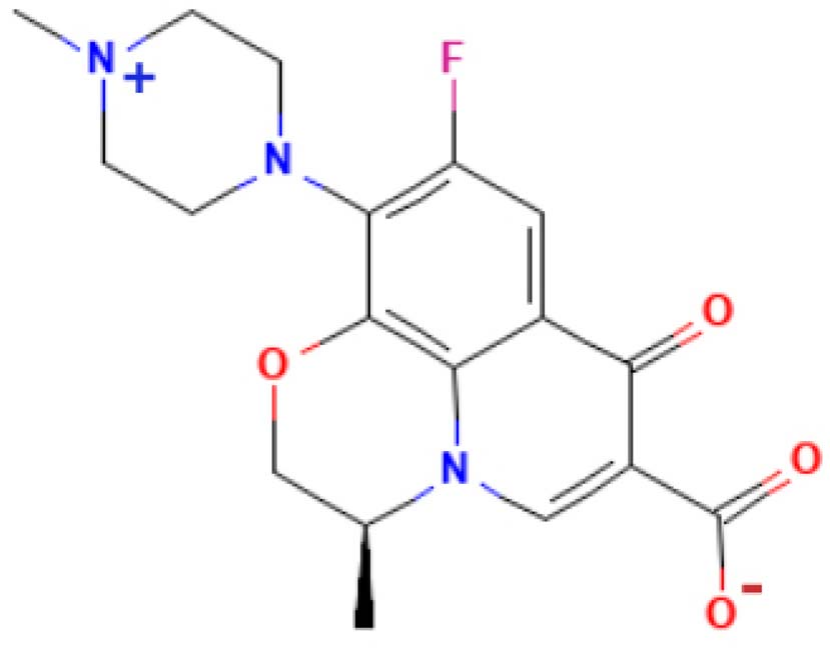
Chemical structure of the amphoteric form of the levofloxacin molecule.

All-atom MD runs of systems were performed using the GROMACS 2021.1 software package.^28^ The construction of all systems was performed using the CHARMM GUI server.^29^ First, the independent components of the system were created using CHARMM GUI options. The levofloxacin molecule (see Fig. 1) forcefield was generated *via* the CHARMM GUI Ligand Reader & Modeler tool. Note that the drug molecule contains a negative charge at the hydroxyl group and a positive charge at the piperazine group, and it is stated that levofloxacin exists as a zwitterionic surfactant in the pH range 5.0–8.5.^30,31^ The anionic (sodium dodecyl sulfate, SDS) and cationic (cetyltrimethylammonium bromide, CTAB) micelles were created using the CHARMM GUI Micelle builder tool with the corresponding 60 SDS and 90 CTAB molecules. The given aggregation numbers for SDS and CTAB micelles are in line with experimental data.^32–34^ After creating the individual components, we generated four systems using the CHARMM GUI Multicomponent Assembler module, which was made from the server's PSF and CRD files. Thus, four independent systems were designed for further simulation:

(a) 30 levofloxacin/60 SDS micelles in water bulk with 150 mM NaCl;

(b) 60 levofloxacin/60 SDS micelles in water bulk with 150 mM NaCl;

(c) 45 levofloxacin/90 CTAB micelles in water bulk with 150 mM NaCl;

(d) 90 levofloxacin/90 CTAB micelles in water bulk with 150 mM NaCl.

The most recent CHARMM36m forcefield^35^ was employed for levofloxacin, SDS, CTAB, and ions; water molecules were modelled using the TIP3P model.^36^ The volume of the simulation box for all cases was set to 10 × 10 × 10 nm^3^.

### MD protocols for all runs

All simulations were carried out according to the following protocol. Systems were subjected to energy optimization (5000 steps) using the steepest descent algorithm. Furthermore, to thermally equilibrate the system, small equilibration runs were performed in both *NVT* and *NPT* ensembles. The minimization and multistep equilibration procedures were done according to CHARMM GUI protocols. Finally, a production run of 1000 ns for each run was performed using the NPT ensemble. As each system was simulated over a long timescale, we did not perform replica statistical simulation runs, as long continuous trajectories are generally considered sufficient for sampling equilibrium micelle–drug interactions.

The target temperature (310.15 K) and pressure (1 bar) were set using the Nosé–Hoover thermostat^37^ and Parrinello–Rahman barostat,^38^ respectively, and temperatures of each species were independently controlled. The bonds were constrained using the LINCS algorithm.^39^ The Particle-Mesh Ewald (PME)^40^ algorithm was used to estimate electrostatic interactions with a cutoff of 1.2 nm. The same cut-off value was also set for van der Waals interactions using a switch function to potentials. The coordinates were written every 0.1 ns, and the timestep of the production run was set to 2 fs.

All production runs were performed on Armenian infrastructure^41^ (“Aznavour” HPC), and the snapshots/visual presentations were generated using the VMD graphical software.^42^ For analysis, we used tools from the GROMACS package and in-house Tcl/Python scripts.

Relative binding energies were computed using g_mmpbsa,^43^ a GROMACS wrapper for the Molecular Mechanics Poisson–Boltzmann Surface Area (MMPBSA) method.^44^ In this method, the binding free energy includes the electrostatic interactions, van der Waals interactions, polar solvation energy, and non-polar solvation energy and can be calculated *via* the following formula:

1 Δ*G*_bind_ = Δ*G*_Complex_ − Δ*G*_Micelle_ − Δ*G*_Drug_

Snapshots from MD trajectories have been used to evaluate the binding energy, and in our case 500 snapshots (500 frames by g_mmpbsa – dt 2000 option) of each complex were selected. We choose 3–4 bound levofloxacin molecules to evaluate the relative binding energy and their corresponding components (vdW and electrostatic) and get their averaged values.

## Results and discussion

### Levofloxacin with SDS micelles

**Fig. 2:**
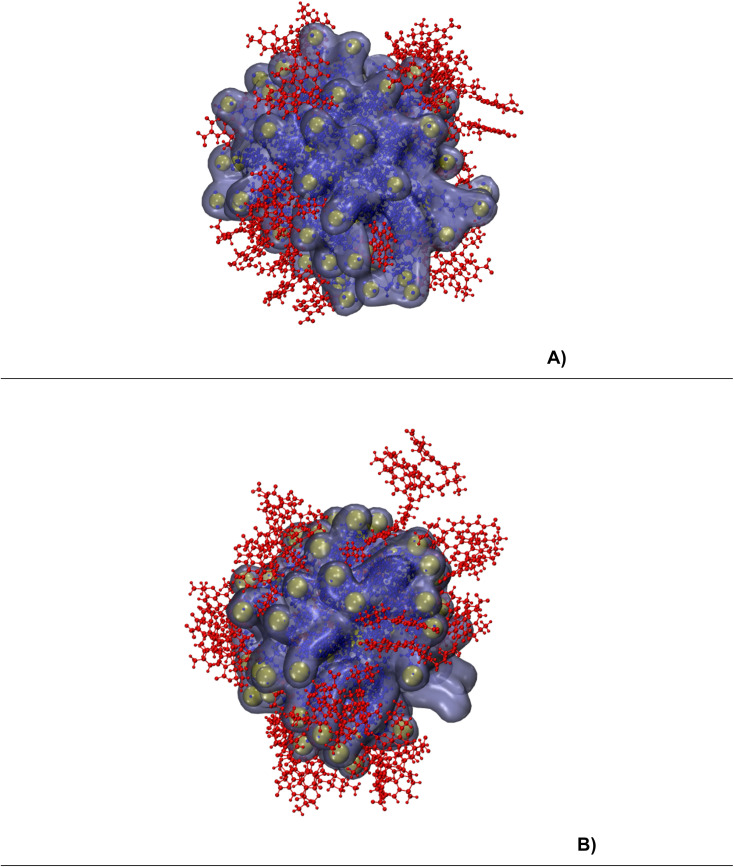
The snapshots from a 1000 ns timepoint for both runs ((A) 30 levofloxacin and (B) 60 levofloxacin). The ions and water molecules are omitted for clarity. Levofloxacin and SDS are colored red and blue, respectively. Sulfur atoms in SDS are colored yellow.

Before calculating the parameters, we provide snapshots (Fig. 2) extracted from the last timestep of trajectories for both runs: 30 levofloxacin molecules with SDS micelles and 60 levofloxacin molecules with SDS micelles.

**Fig. 3:**
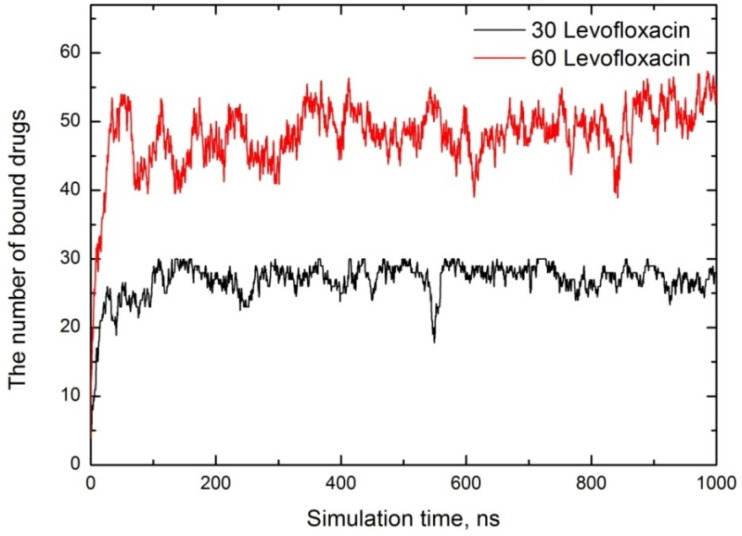
The number of encapsulated drug molecules depending on simulation time for both cases.

First, to estimate the loading capacity of micelles, we calculated the number of trapped or captive drug molecules depending on the simulation time, and the drug-binding affinity plot for both runs is shown in Fig. 3. Note that we consider a bound state if the cut-off distance between the COM of the piperazine group of drug and the surfactant headgroup is less than 8 Å.

As is shown, at a low drug concentration (30 levofloxacin), almost all molecules are attached to or detached from the micelle, and the mean number is estimated to be ∼27. With a twofold increase in drug concentration, we observe that molecules still tend to bind to the SDS micelle. In this case, the maximum number of bound drugs is 58, while the average number is approximately 48. The drug loading capacity (DLC (wt%)) and entrapment efficiency (EE (%)), typically expressed as the ratio of the number of encapsulated drugs to the number of micelles, are essential parameters for evaluating the pharmacokinetics of small species. These definitions follow the standard conceptual description of DLC and EE widely used in drug-delivery studies. Consequently, in the SDS micelle system, the 60 levofloxacin/60 SDS micelle case, the maximum drug loading was 54.8 wt% (average: 50.1 wt%) and the maximum entrapment efficiency was 96.7% (average: 80%). Unfortunately, there is no experimental data for direct comparison; however, one can assume that the drug-loading capacity was higher than that of other reported systems, such as levofloxacin-loaded chitosan nanoparticles with entrapment efficiencies ranging from 57.14% to 87.47% and loading capacities from 15 wt% to 25 wt%.^45,46^ Levofloxacin analog ciprofloxacin displays an entrapment efficiency of ∼54.11% when dealing with poly-ε-caprolactone (PCL) nanoparticles, while levofloxacin-loaded polymeric nanoparticles using PCL are entrapped less efficiently (28.14%).^47^ The study by Beraldo-Araújo *et al.*^48^ shows that levofloxacin encapsulated in nanostructured lipid carriers has an entrapment efficiency of 71.9% to 78.9%, as determined by Differential Scanning Calorimetry (DSC) and X-ray diffraction analysis.

**Fig. 4:**
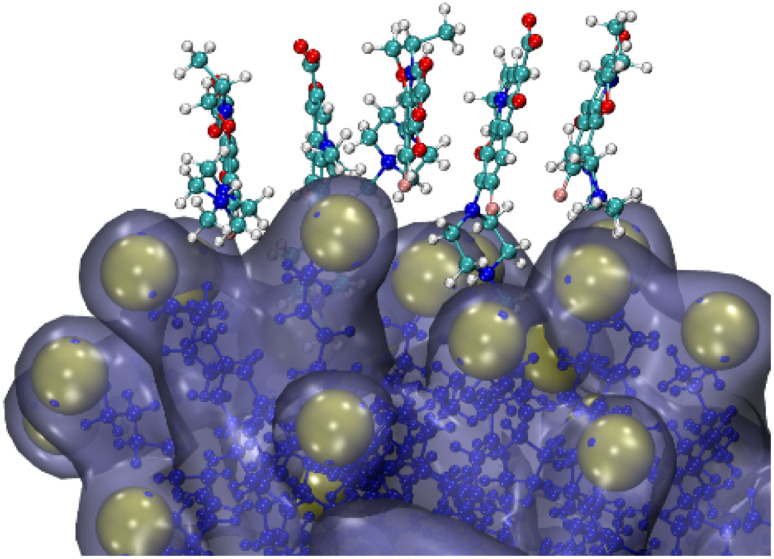
Fragmental snapshot of a 1000 ns run (higher concentration – 60 levofloxacin/60 SDS).

It is interesting to note that, depending on the levofloxacin concentration, 5 to 8 molecules of levofloxacin form a stacked aggregate (see a fragment of a snapshot in Fig. 4), and the columnar stacks are not stable. We tracked that during the simulation time, the stacks ruptured, where the lifetime is roughly ∼50–100 ns. Probably, such a kind of mutual orientation where the levofloxacin rings align face-to-face (“sandwich” orientation) can be driven by attractive forces between their pi-electron clouds. A similar effect was reported^49^ for the zwitterionic ciprofloxacin on the lipid membrane, where the drug molecules approach the membrane in stacks.

**Fig. 5:**
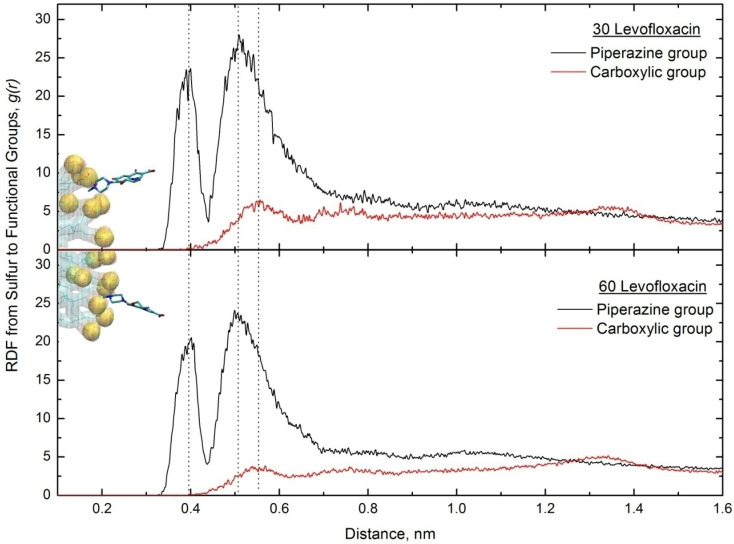
The SDS sulfur-to-levofloxacin functional group for both low and high drug concentrations. The fragments of snapshots from the last time point of both simulation runs are also embedded. A randomly selected levofloxacin molecule was used to show the orientation.

To understand the orientation of levofloxacin, we have determined the radial distribution function of levofloxacin's two functional charged groups (carboxylic and piperazine) from SDS sulfurs, as the visual inspection of trajectories of both runs indicates that most of the drug molecules' piperazine amine groups are strongly oriented towards the SDS headgroup. The RDF plots are monitored in Fig. 5.

Discussing the orientation of drug molecules, we observe two sharp peaks at ∼0.4 nm and 0.5 nm for the charged piperazine group, indicating a strong interaction between the sulfur atoms. In contrast, the peak for the oxygen group appears at ∼0.55 nm, indicating that the levofloxacin molecule is oriented in such a way that the charged piperazine group is in close contact with SDS sulfur. This suggests that SDS micelles interact exclusively with the drug's charged regions.^15^ Shakeel and coworkers^50^ studied levofloxacin/SDS micelles using surface tension and absorbance measurements, demonstrating a strong interaction between levofloxacin with SDS. They conclude that the drug molecules are located on the surface of the micelle. Acharya and coworkers,^22^ while discussing the adsorption mechanisms of a similar fluoroquinolone antibiotic (ciprofloxacin) on SDS bilayers, claim that stronger interactions between the hydrophilic head of the surfactant and the ciprofloxacin drug result in the drug molecule being localized near the SDS headgroup. They also argue that this localization is due to the presence of a hydrogen bond interaction between ciprofloxacin and SDS.

**Fig. 6:**
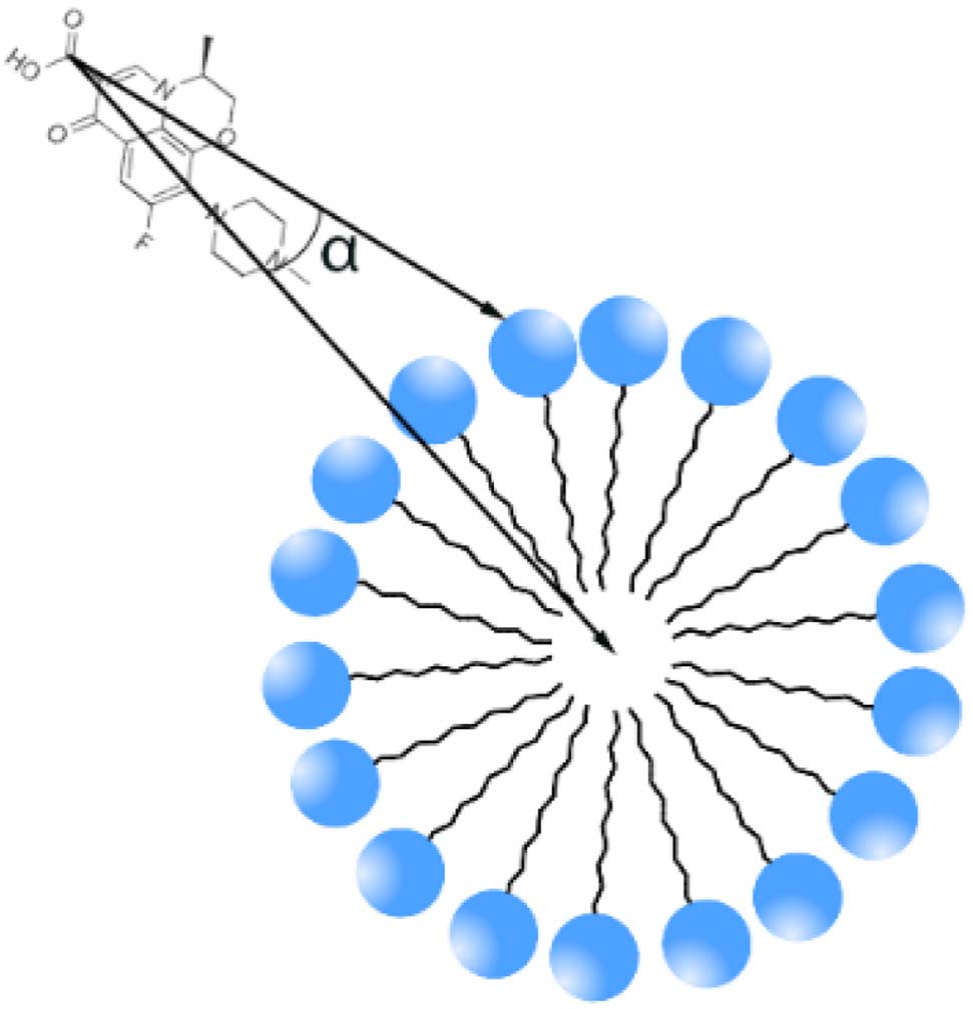
The schematic presentation of an angle.

**Fig. 7:**
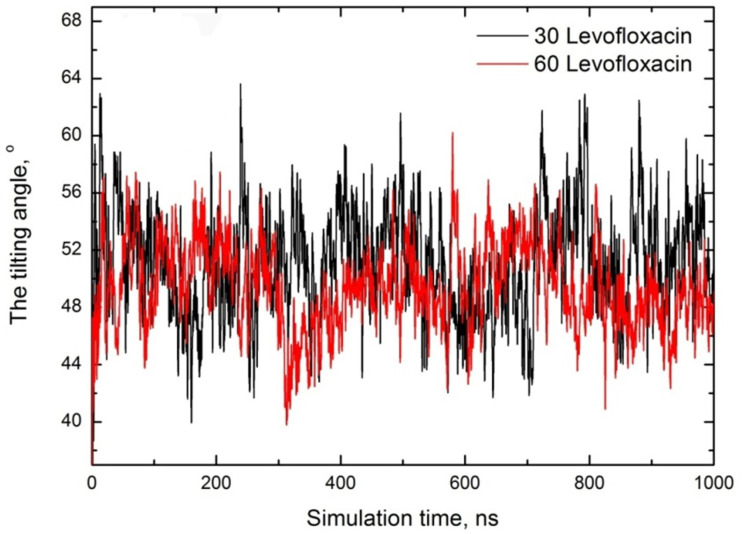
Orientational angle of levofloxacin depending on simulation time for both cases. A picture schematizing the angle definition is also embedded for clarity.

To quantify the orientational preference of levofloxacin molecules near the SDS micelle interface, we calculated the angle between two vectors: one is the vector from levofloxacin carboxyl to piperazine group, and the second is the vector from levofloxacin carboxyl to the SDS micelle center of mass (COM) (see Fig. 6). Note that only levofloxacin molecules with their piperazine group within 1 nm of any SDS sulfur were included, *i.e.* we have only calculated the tilting angle of encapsulated levofloxacin molecules. This orientation angle provides a measure of levofloxacin drug tilt relative to the micelle radial axis: ∼0° indicates the drug points toward the micelle COM, ∼90° suggests a tangential orientation, *i.e.* parallel to the surface and ∼180° implies an outward orientation. The plot for both cases is monitored in Fig. 7.

The observed mean angle (averaged over the last 200 ns of run) at a small concentration is estimated to be ∼51°, which shows that levofloxacin adopts a tilted orientation near the SDS headgroup by balancing interactions between the levofloxacin positively charged piperazine group and SDS headgroup sulfurs. The twice increase of the levofloxacin concentration (drug amount/SDS molecules = 1) leads to a slight increase in the tilting angle, and the mean angle is estimated to be ∼48°, which suggests enhanced packing of drug molecules.

**Fig. 8:**
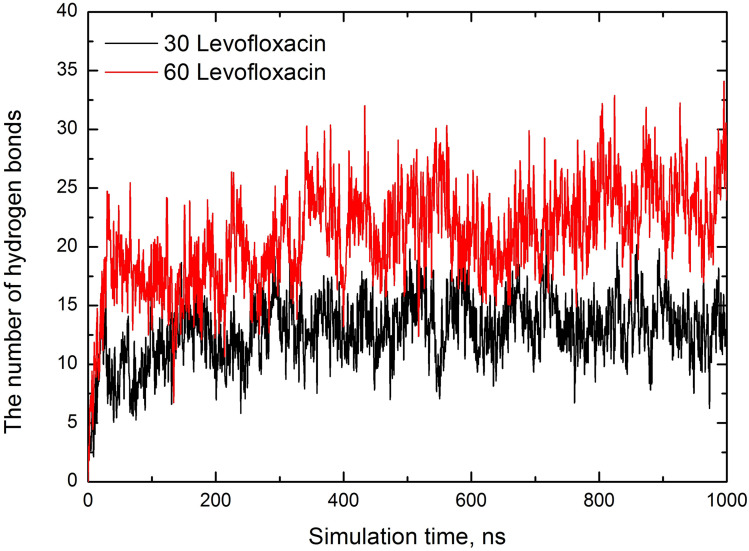
The number of hydrogen bonds depending on the simulation time for both series of runs.

To confirm the existence of hydrogen bonding in the levofloxacin–SDS complex, the trajectories were analyzed *via* the GROMACS hbond module. Note that the hydrogen bonds were defined using the standard criteria, where the donor–acceptor distance is less than 0.35 nm and the hydrogen-donor–acceptor angle is less than 30°. The number of hydrogen bonds throughout the simulation is shown in Fig. 8.

We observe that the number of hydrogen bonds increases over time during the simulation, and at the end of the simulation up to half of the levofloxacin molecules are hydrogen-bonded to the SDS headgroup oxygen *via* the levofloxacin piperazine hydrogen, thereby enhancing the stability of the levofloxacin–SDS interactions. We have examined the hydrogen bond network between water and levofloxacin (see Fig. 1S in SI), as hydrogen bonds have a fundamental contribution to stabilize zwitterions in water,^51–53^ suggesting a solvation of the carboxylate −COO^−^ group by roughly 3–5 water molecules.^54^ Analyzing the water–levofloxacin hydrogen network, we argue that, on average, 7–8 water contacts per drug are available.

### Levofloxacin with CTAB micelles

**Fig. 9:**
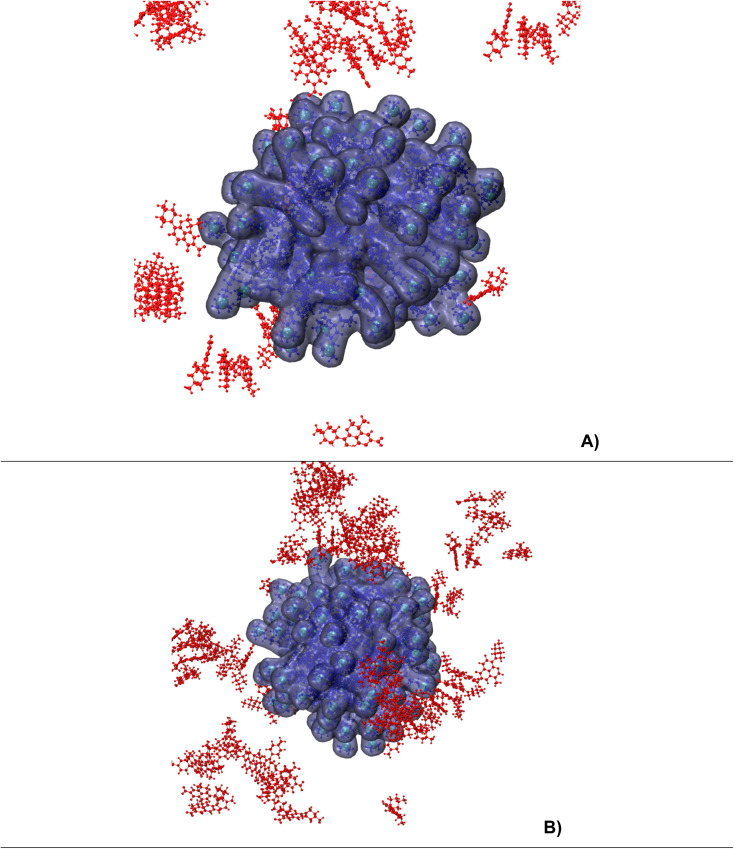
The snapshots from a 1000 ns timepoint for both runs (A – 45 levofloxacin; B – 90 levofloxacin). The ions and water molecules are omitted for clarity. Levofloxacin and CTAB molecules are colored in red and blue, respectively. Nitrogen in CTAB is shown in cyan.

For CTAB, before doing the analysis, we also provide the snapshots (Fig. 9) extracted from the last time point of trajectories for both runs: 45 levofloxacin molecules with CTAB micelles and 90 levofloxacin molecules with CTAB micelles.

**Fig. 10:**
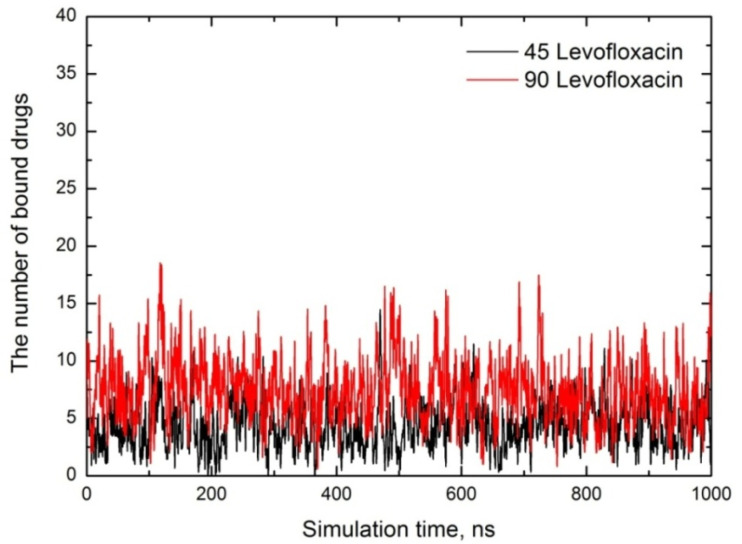
The number of encapsulated drug molecules depending on the simulation time for both cases.

To estimate the number of bound levofloxacin molecules, we apply the same code for calculation, and both curves are shown in Fig. 10.

Here, in the case of CTAB micelles, one can assume that the concentration of levofloxacin has little effect on the number of accumulated drugs, and the average number of trapped drug molecules are ∼5 (∼11% of total) and ∼7 (∼8% of total) for both cases, respectively, *i.e.* one can argue that a small amount of levofloxacin molecules tend to bound to CTAB micelles; meanwhile, when dealing with SDS micelles, about 80–90% of levofloxacin molecules are bound to the micelle. Analysis of the loading capacity and entrapment efficiency shows that for 90 levofloxacin/90 CTAB micelles, the maximum drug loading was 15.8 wt% (average: 7.2 wt%) with a maximum entrapment efficiency of 18.5% (average: 7.8%). For direct comparison, we provide the MD data from ref. 23, where a very short (2 ns) MD simulation of six levofloxacin molecules with 32 CTAB micelles was performed in the presence and absence of salt. According to ref. 23, after 2 ns of run, all six levofloxacin molecules were trapped by the CTAB spherical micelle, and the loading capacity was estimated to be ∼15.67%. At the same time, the entrapment efficiency was 100%. The loading capacity value aligned with our MD results; however, the efficiency was overestimated due to the very short simulation time. Using X-ray diffraction and thermal gravimetric analysis (TGA), Cui and coworkers^55^ quantified the levofloxacin loading capacity. They synthesized twisted rod-like chiral mesoporous silica materials (using CTAB as a template) with achiral alcohols and co-structure-directing agents. According to results, the loading capacity was up to 94.57%.^55^ Moreover, examining the adsorption properties of some fluoroquinolone antibiotics at the gas–liquid interface using a foam fractionation process, Ghosh and coworkers^18^ argue that fluoroquinolones (levofloxacin, ciprofloxacin, ofloxacin and norfloxacin) show a higher removal efficiency with SDS (91.7%, 96.3%, 96.7% and 97.9%) compared with CTAB, which is in the range of 52%, *i.e.* one can assume that CTAB–fluoroquinolone complexes are poorly adsorbed at the gas–liquid interface. We suggest that pure CTAB may have low efficiency for levofloxacin delivery without formulation optimizations, *e.g.* considering some additives or mixing with nonionic amphiphilic surfactants^56^

**Fig. 11:**
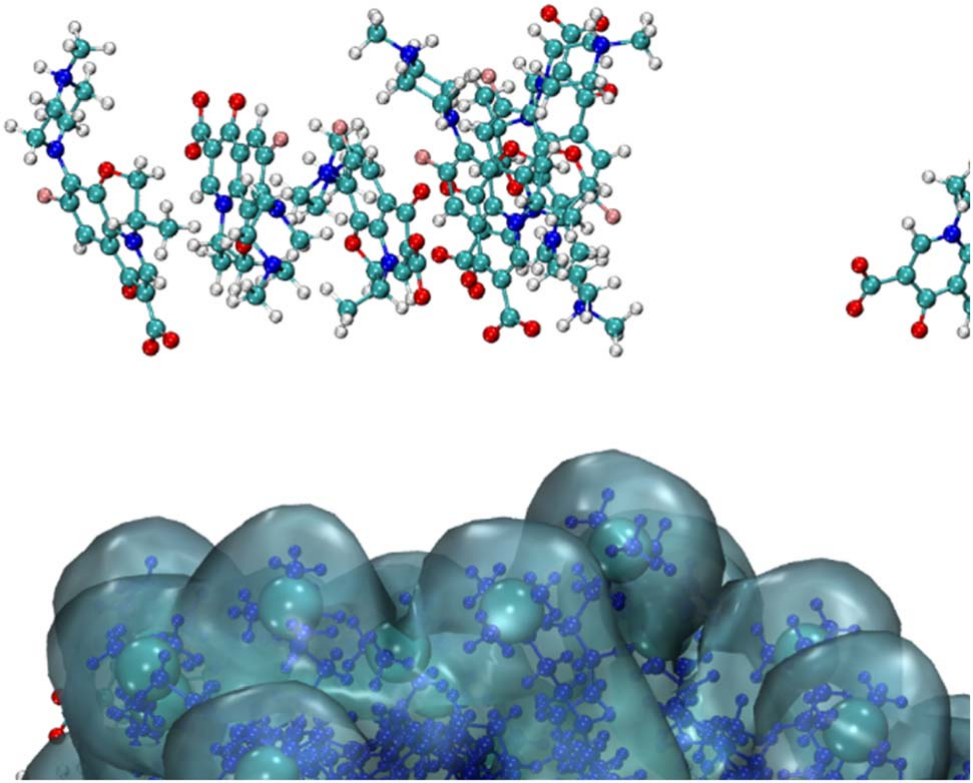
Fragmental snapshot of a 365 ns run (higher concentration – 90 levofloxacin/90 CTAB).

It is interesting to note that in the case of CTAB micelles, we also track columnar stacks as in the case of SDS; however, these stacked aggregates are far from CTAB micelles (see Fig. 11). Due to π–π interactions and hydrogen bonding,^57^ the stack formation in water^49^ as well as in crystalline form^58^ is reported by many authors, and in the case of CTAB micelles, we track that the fluoroquinolone molecules formed and broke (detached) in water bulk over the entire process of the production run.^51^

**Fig. 12:**
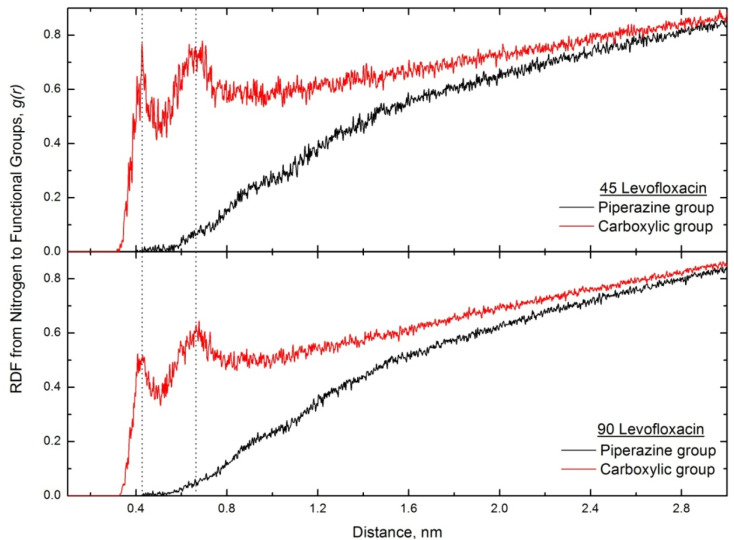
The CTAB nitrogen to levofloxacin functional group RDFs for both low and high drug concentrations.

Similar to the SDS case, for levofloxacin/CTAB systems, we have determined the radial distribution function of levofloxacin's two functional charged groups (carboxylic and piperazine) from CTAB nitrogens; however, the visual inspection of trajectories of both runs indicates that most of the captured drug molecules' carboxylic groups are oriented towards the CTAB headgroup, while those of the piperazine group are directed to water bulk, *i.e.* we track the levofloxacin orientation in comparison to the SDS case. The corresponding RDF plots are shown in Fig. 12.

As expected, we track two peaks at ∼0.41 nm and ∼0.67 nm for the carboxylic group, where the levofloxacin molecule is oriented in such a way that the negatively charged carboxylic group is in contact with the CTAB nitrogen, *i.e.*, in terms of orientation, we see the reverse picture as compared with SDS micelles. We found that a small number of bound levofloxacin molecules are oriented towards the micelle headgroup, where the carboxylate group is –COO^−^.

**Fig. 13:**
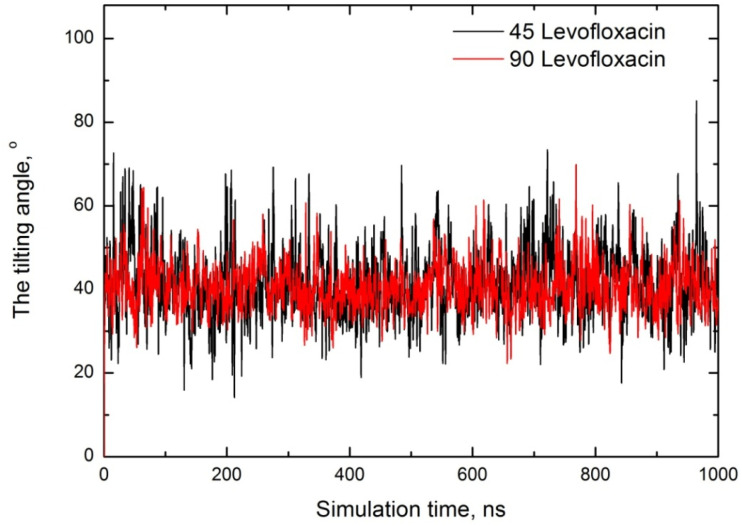
Orientational angle of levofloxacin depending on the simulation time for both cases.

To verify the orientation of trapped levofloxacin molecules, we have determined the following angle between two vectors: one is the vector from levofloxacin piperazine to the carboxyl group, and the second is the vector from levofloxacin piperazine to the CTAB micelle COM. As can be seen, this is the same angle as discussed below (*vice versa*). Note that, in this case, we have included the levofloxacin molecules, where the carboxylic-to-CTAB COM distance is less than 1 nm, *i.e.* we have calculated the tilting angle of encapsulated levofloxacin molecules only. All graphs are shown in Fig. 13.

Examining the orientation angle, we see that at low concentrations, the average angle is ∼42°, suggesting that levofloxacin adopts a tilted orientation near the CTAB headgroup. The twofold increase in the levofloxacin concentration does not lead to significant differences, and the averaged angle is estimated to be ∼40°.

The calculation of the hydrogen bond network between CTAB and levofloxacin molecules using the GROMACS hbond module shows that there are no hydrogen bonds. In contrast, levofloxacin forms hydrogen bonds with water molecules (see Fig. 2S in SI).

### Binding energies between drugs and micelles

To gain deeper insights into drug encapsulation by micelles, relative binding free energy calculations, including their components, were performed between the drugs and the micelles. For SDS micelles, drugs solubilized within the micelle core were selected, while for CTAB micelles, drugs in closest proximity to the micelle surface were used in the analysis. The resulting mean values of interaction energies, including van der Waals, electrostatic, and relative binding energies for all cases, are shown in Table 1.

**Table 1:** The average relative binding energies (vdW and electrostatic components) between drugs and the micelle for each system

Systems	vdW component (kJ mol^−1^)	Electrostatic component (kJ mol^−1^)	Relative binding energy (kJ mol^−1^)
45 drug/90 CTAB micelles	−0.81785	−68.8891	−71.5802
90 drug/90 CTAB micelles	−0.5168	−58.8052	−63.8747
30 drug/60 SDS micelles	−52.2017	−323.309	−195.642
60 drug/60 SDS micelles	−35.1645	−334.909	−201.821

The values of relative binding energy for all cases were found to be negative, which signifies the formation of thermodynamically stable complexes. Note that the energies were calculated from the last 100 ns of the simulation run for all cases. For SDS systems, the relative binding free energy calculated *via* the g_mmpbsa module shows Δ*G* in the range of −195 kJ mol^−1^ to −201 kJ mol^−1^, where the non-bonded van der Waals interactions and electrostatic interactions are in the range of −35 kJ mol^−1^ to −52 kJ mol^−1^ and from −323 kJ mol^−1^ to −334 kJ mol^−1^, respectively; meanwhile, for CTAB systems, relative binding energies (from ∼63 kJ mol^−1^ to −71 kJ mol^−1^) and their components are significantly lower than SDS, *i.e.* comparing the SDS and CTAB systems, we observe that the drugs bind more favorably to the SDS-based micelles. In contrast, for the CTAB-based micelles, the relative value of the binding energy is significantly lower.

Discussing energetic aspects of levofloxacin in water in the presence of SDS, Shakeel *et al.*^50^ calculated the binding constant and free energy of binding at 298 K, and accordingly the binding energy Δ*G*_bind_ = −26.99 ± 0.02 kJ mol^−1^ was obtained, which is not comparable with MD data, as the g_mmpbsa model does not include entropy calculation (−*T*Δ*S*) due to the expensive computational cost of normal mode analysis (NMA).^59^

## Conclusion

Recently, surfactant-based drug delivery nanocarriers have garnered considerable attention due to their high drug solubility, biocompatibility, chemical stability, and targeted delivery, thereby reducing side effects. In this regard, we have carried out a series of long-scale MD simulation runs (1000 ns of each case) to figure out the orientation and dynamic feature of fluoroquinolone antibiotic (levofloxacin) within anionic SDS and cationic CTAB micelles.

A long-scale MD simulation shows that levofloxacin can be entrapped more effectively within SDS micelles than within the CTAB-based ones. In comparing the drug-loading capacity and entrapment efficiency, we show that in the case of SDS, the entrapment efficiency is almost ten times higher than that in CTAB micelles. Furthermore, the loading capacity for SDS micelles is 54.8 wt%, while for the CTAB micelles, it is ∼15.8 wt%. Hence, we suggest that CTAB micellar systems, despite strong molecular interactions, exhibit lower binding strength and reduced loading efficiency for levofloxacin drug delivery.

Discussing the binding mechanism, we see that in the case of SDS micelles, levofloxacin's positively charged piperazine group, which is oriented towards the SDS headgroup sulfurs, is capable of binding to the SDS's charged headgroup, and apart from the electrostatic interaction between charged species, the presence of a hydrogen bond network between SDS and levofloxacin leads to additional stability of levofloxacin–SDS interactions. Therefore, in levofloxacin-loaded SDS micelle systems, electrostatic and H-bonding interactions are available for stabilization. In contrast to the SDS system, in the levofloxacin/CTAB system, no hydrogen bonding is observed, while levofloxacin drug molecules adsorb onto the micelle surface. This indicates that the levofloxacin carboxylic groups are oriented towards the CTAB headgroup. It should also be noted that less than 10% of levofloxacin molecules solubilize in CTAB micelles, and more than 90% of drug molecules remain unentrapped (in the water phase).

The relative binding energy calculation, which predicts the strength of drug with micelles, shows that the levofloxacin–SDS micelle complex is more stable than that of the CTAB micelle, *i.e.* levofloxacin shows a higher binding affinity to SDS micelles as compared to CTAB.

## Conflicts of interest

The authors declare that they have no conflict of interest.

## Supplementary Material

NA-OLF-D5NA00884K-s001

NA-OLF-D5NA00884K-s002

## Data Availability

Some figures (Fig. S1 and S2) and coordinate files (last frame .gro files) presented in the study are included in the supplementary information (SI), while further inquiries can be directed to the corresponding author. Supplementary information is available. See DOI: https://doi.org/10.1039/d5na00884k.
