# Supplementary figures and images for: Levofloxacin-loaded surfactant nanocarriers: a computational study
Source: Nanoscale Adv. 2026 Jan 24;8(5):1648–60. doi: 10.1039/d5na00884k (PMC12875406; doi:10.1039/d5na00884k)

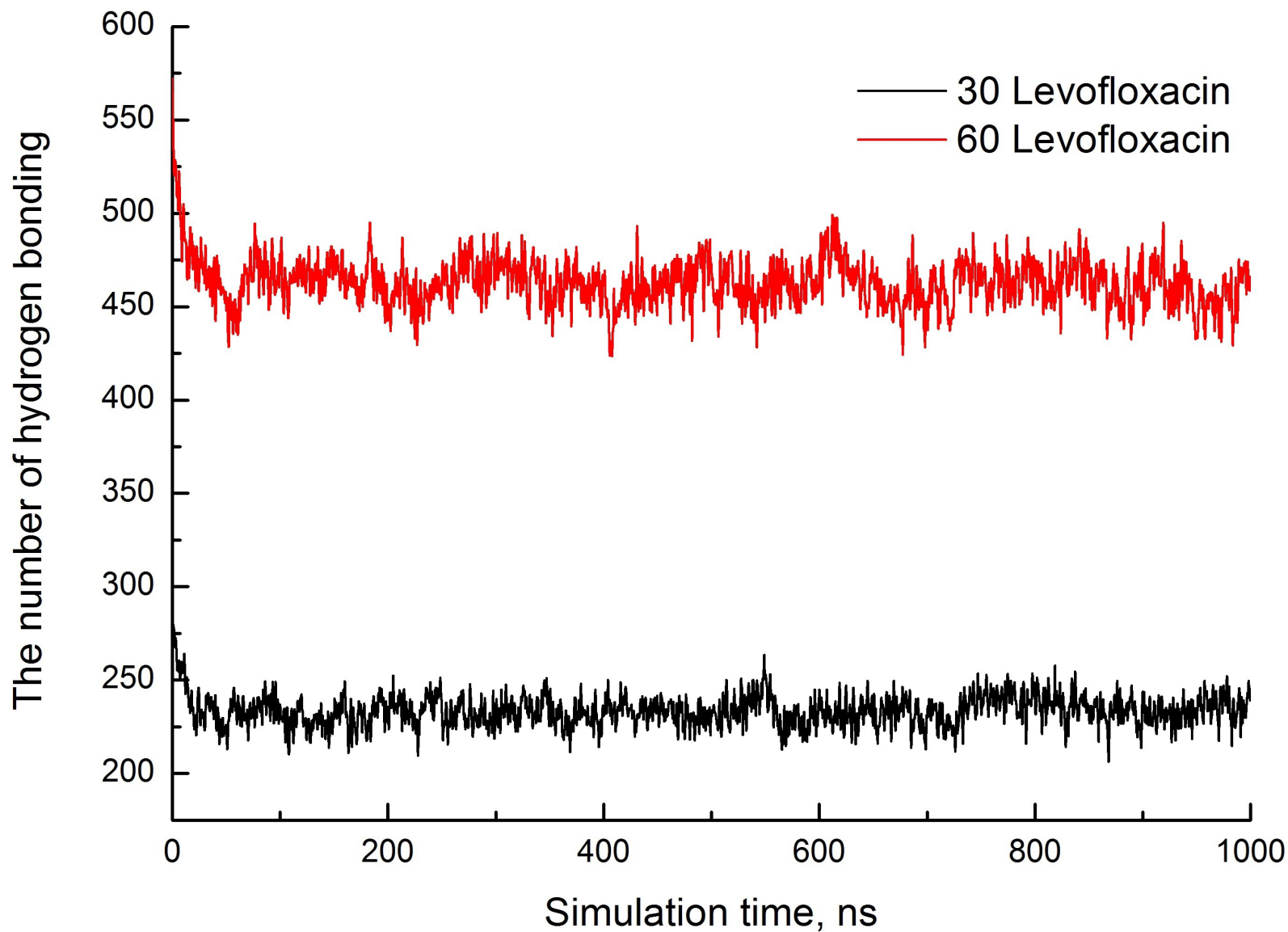

Supplement: NA-008-D5NA00884K-s001 [file NA-008-D5NA00884K-s001.pdf]

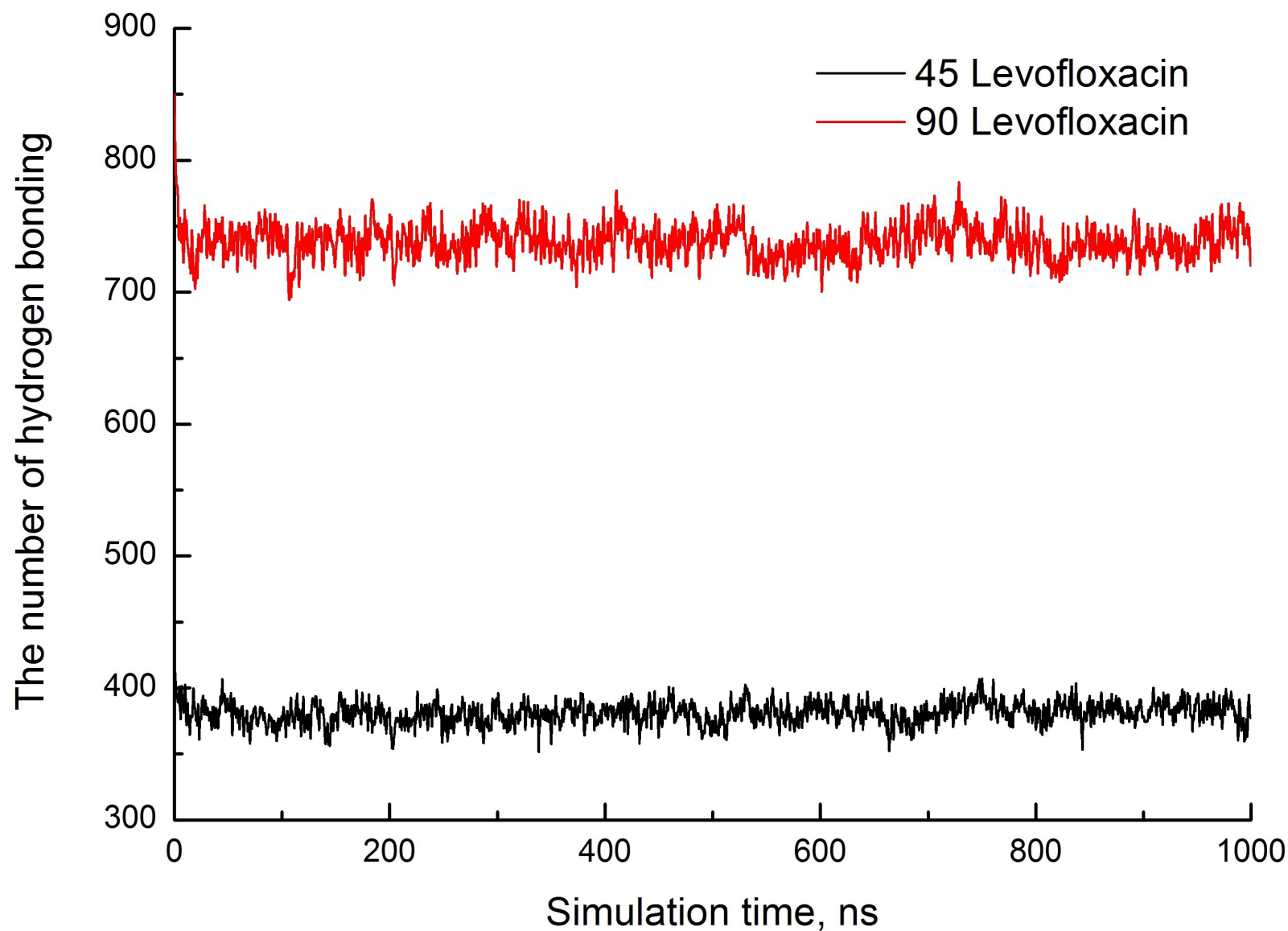

Supplement: NA-008-D5NA00884K-s002 [file NA-008-D5NA00884K-s002.pdf]
